# Pectinesterase activity and gene expression correlate with pathogenesis of *Phytophthora infestans*

**DOI:** 10.3389/fpls.2024.1481165

**Published:** 2024-11-12

**Authors:** Linmei Deng, Xun Huang, Jian Dao, Yajin Xu, Kunyan Zhou, Wenping Wang, Chunjiang Liu, Meng Chen, Shunhong Zhang, Yue Zhang, Jianjun Hao, Xia Liu, Yanli Yang

**Affiliations:** ^1^ Key Laboratory for Agro-biodiversity and Pest Control of Ministry of Education, College of Plant Protection, Yunnan Agricultural University, Kunming, China; ^2^ School of Food and Agriculture, The University of Maine, Orono, ME, United States

**Keywords:** potato late blight, pathogen-host interaction, gene expression, physiological race, cell wall degrading enzyme

## Abstract

Late blight caused by *Phytophthora infestans* is the most devastating disease of potato. *Phytophthora infestans* produces many secondary metabolites and effector proteins, involved in the pathogenesis, which compromise host defense mechanisms. Pectinesterase (PE) is a cell wall degrading enzyme secreted by *P. infestans* to infect the host. To examine the role of PE in *P. infestans*, 15 strains of *P. infestans* were isolated from infected potato leaves in Yunnan, China. We analyzed the biological effects of exogenously added PE on *P. infestans* and its activity and gene expression after infection of potato using quantitative real-time polymerase chain reaction (RT-PCR). It was found that PE significantly promotes the growth of *P. infestans*, increases the weight of mycelium and the number of sporangia, and promotes the sporangial germination. PE accelerated the infection process of *P. infestans* on potato. The pathogenicity of *P. infestans* was positively correlated with PE activity and gene expression. PE is a key to the virulence difference of potato late blight.

## Introduction

Potato (*Solanum tuberosum*) is one of the three top food crops in the world (Naumann et al., 2020). Due to its short growth period and strong adaptability to the environment, it has been cultivated in more than 120 countries and regions in the world. According to the Food and Agriculture Organization of the United Nations (FAO) (http://www.fao.org/), in 2021, the world’s potato planted area was more than 18 million hectares, with a total yield of 376 million tons, of which China ranked the first. Late blight caused by *Phytophthora infestans* is the most devastating disease of potato. At present, the use of chemical products and the cultivation of disease-resistant varieties are commonly used and effective means to control potato late blight (Najdabbasi et al., 2022; Rogozina et al., 2021). However, due to the adaptability of *P. infestans* to environmental changes, new physiological races are continuously evolving, resulting in the loss of variety resistance and difficulty in disease control (Ivanov et al., 2021). *Phytophthora infestans* produces many secondary metabolites and effector proteins to deactivate the host’s defense while infecting the host, thereby promoting infection (Yang et al., 2021; Li et al., 2020), which is common in many other plant pathogens (Kubicek et al., 2014).

Pectinase is an enzyme complex that consists of several different types of enzymes that collectively act on pectin, a polysaccharide found in plant cell walls. The primary compounds or types of enzymes that make up pectinase includes polygalacturonase, pectin methylesterase, and pectin lyase. These compounds are cell-wall-degrading enzymes secreted by plant pathogens during host infection and are pathogenicity factors (Bravo et al., 2016). Pectinesterase (PE) is one of the crucial cell wall degrading enzymes secreted by plant pathogens, including pathogenic fungi, oomycetes and bacteria (Xue et al., 2018). In 1979, Krátká and Veselý (1979) first detected the activity of PE from *Pythium ultimum*, *Pythium oligandrum* and *Pythium debaryanum*. Several decades later, Ospina-Giraldo et al. (2010) found highly complex CAZy homologs in the genomes of *P. infestans*, *P. sojae*, and *P. ramorum*. A large number of CAZy homologs play an important role in pathogenicity by participating in the degradation of plant cell walls. respectively, of which pectinase accounted for about 25% (Blackman et al., 2014). Since then, this gene has been further investigated in it roles. Pectinesterase has also been studied in *P. infestans*, it can contribute to the breakdown of plant tissues, which can facilitate infection by degrading pectin (Sabbadin et al., 2021). Research into pectinesterase in *P. infestans* often focuses on understanding how the enzyme helps the pathogen invade and colonize host plants. The enzyme’s activity can be crucial for the pathogen’s ability to overcome plant defenses and establish an infection, but requires further investigations. The objective of this study was to investigate the effects of exogenous addition of PE on *P. infestans* and its infection of potato, and to clarify the role of PE activity and expression in the pathogenesis of *P. infestans*. The outcome would provide foundation for understanding plant-host interactions and advancing plant breeding efforts.

## Materials and methods

### Effects of PE on *P. infestans in vitro*

A total of 15 strains of *P. infestans* with different pathogenicity were isolated from potato leaves showing late blight symptoms in Yunnan, China (**Table 1**). The pathogenicity data in the table were determined according to method of detached-leaf inoculation (Raza et al., 2019) in the early stage. For pathogen inoculation, compound leaves from the top to the third leaf position were chosen from potato ‘S88’ grown for 45 days. PE (30,000 U/g, Yien Chemistry, Shanghai, China), derived from *Aspergillus niger*, was initially dissolved in 99.7% ethanol subsequently diluted with ddH_2_O to achieve a final concentration of 1,000 U/mL PE, containing 0.1% ethanol, which was used as a stock solution for later use.

**Table 1:** Measurement of disease lesion on potato leaves inoculated with different strains of *Phytophthora infestans*.

Strain	Location of isolation	Virulence	Lesion area (%)
HS03	Huize	Weak	3.77 ± 4.25h
JCZ29	Huize	Weak	10.42 ± 3.67gh
HZ02	Huize	Weak	12.66 ± 4.90g
HS02	Huize	Weak	19.79 ± 3.86f
DSPQ006	Dali	Medium	20.04 ± 3.28f
MLS13418-2	Malong	Medium	23.79 ± 7.9f
DQ01	Diqing	Medium	38.46 ± 6.89e
MLS8802	Malong	Medium	48.49 ± 7.36d
NLL605	Lijiang	Medium	56.27 ± 3.17c
HJG02	Dali	Strong	60.43 ± 5.22c
LJ04	Lijiang	Strong	61.49 ± 6.08c
XDL601	Xundian	Strong	64.36 ± 16.65c
ZTQ907	Zhaotong	Strong	70.63 ± 10.42b
DL04	Dali	Strong	76.26 ± 5.66b
ML01-2018	Malong	Strong	90.10 ± 6.44a

Different letters indicate significant differences between treatments (p < 0.05).

In a petri plate, rye tomato agar (Qian et al., 2021) was amended with PE stock solution at series of concentrations including 0, 10, 100, and 1,000 U/mL. Four strains of *P. infestans* (HZ02, DL04, HJG02, and ML01-2018) was inoculated onto the plate and grown for 8 days of incubation at 19 °C in the dark. The colony diameter was measured perpendicularly on days 4, 6, and 8. Mycelia were scraped off from the plate and weighed, and then homogenized with water. Sporangia were counted using a hemocytometer. According to the method of Zhu et al. (2015), DL04 sporangia suspension was mixed with different concentrations of PE solution at a ratio of 1:1 to prepare a sporangia suspension containing 8,000 sporangia per mL and various concentrations of PE. Five microliters were pipetted onto the concave slide, and the cover glass was placed in a dark incubator at 19°C for 48 h. Both zoosporic and sporangial germinations were observed under a biomicroscope (E200MV, Nikon, Nanjing, China). Each treatment contained three replicates.

### Effect of PE on *P. infestans* in planta

The mycelia of *P. infestans* DL04 and HJG02 cultured for 10 days were collected using toothpicks, and ground with sterilized water in a mortar with pestle. The sporangium was obtained by filtration through a 300-mesh filter. The sporangia were transferred into 100 U/mL or 1,000 U/mL PE solutions and adjusted to a concentration of 8,000 sporangia per mL, which were used as inocula. According to method of detached-leaf inoculation (Raza et al., 2019), compound potato leaves were cut from the third leaf position and washed three times using sterilized water. Potato tubers were cut into one centimeter disks. Each leaf and tuber was a replicate with six replicates per treatment. Both the leaves and tuber disks were placed on 0.8% water agar. The *P. infestans*-PE mixture (25 μL) was inoculated onto both sides of the leaf or the center of the tuber disk. Inoculation of sporangium suspension was used as control. One day after the inoculation, the leaves were flipped over and placed in an incubator set to a 12 h light/dark cycle at 19 °C, along with the tuber disk. Disease was evaluated three days after the treatment. The ratio of leaf and tuber lesion area to total leaf and tuber area was used to evaluate the incidence of the disease.

### Measurement of PE activity

Enzymatic analysis was performed using a pectinesterase kit (Suzhou Grace Biotechnology Co., Ltd., Suzhou, China) to determine the enzymatic activity of *P. infestans* strains and infected potato leaves. Briefly, 0.15 g *P. infestans* mycelia were ground with 1.5 mL of the pectinesterase kit in a mortar with a pestle in an ice bath and centrifuged at 12,000 g at 4°C for 15 min. An aliquot of 1 mL of the supernatant was transferred into a centrifuge tube, and 25 μL of reagent 2 and 4 mL of reagent 1 were added in order and mixed well. Subsequently, reagent 3 was added to adjust pH to 7.8 (pink). The tubes were incubated at 37°C in a water bath for 60 min. The pH was maintained to 7.8 (pink) with reagent 4 every 20 minutes. At the same time, the volume V2 (mL) of the consumed reagent 4 was recorded. PE activity (μmoL/min/g) was calculated as = 30 × V2 ÷ W × D (V2: the amount of reagent 4 consumed by titration; D: sample dilution factor; W: sample weight) (Wang et al., 2023).

### Cloning and real-time absolute quantification of the PE gene *pipme1* in *P. infestans*

The DNA of each *P. infestans* strain was extracted using the Ezup Column Fungal Genomic DNA Extraction Kit (B518259, Sangon Biotech, Shanghai, China). DNA quality was examined on a 1.5% agarose gel using electrophoresis. Pectinesterase nucleotide sequence (XM_ 002907416.1) was used as a template. Polymerase chain reaction (PCR) was performed using the primer pair for *pipme1*: Pipme1-F1 (5’ CGGTGTCGGAAGGGGTAG 3’) and Pipme1-R1 (5’ TAAGCAGCAGCGTGGTCG 3’). The PCR reaction was prepared in a 25 μL system, containing 2 μL of 10X PCR buffer, 0.5 μL of primer F (10 μM), 0.5 μL of primer R (10 μM), 0.5 μL of dNTP (10 mM), 2 μL of MgCl2 (25 mM), 0.5 μL of Taq Plus DNA Polymerase (5 U/μL), 2 μL of DNA, and 17 μL of ddH_2_O. The thermal cycler conditions were as follows: pre-denaturation at 95°C for 3 min, 35 cycles of denaturation at 95°C for 30 s, annealing at 57°C for 30 s, and extension at 72°C for 30 s, followed by a final extension at 72°C for 8 min. The PCR products were examined through 1.5% agarose gel using electrophoresis and the target band was cut off and recovered with SanPrep column DNA gel recovery kit (B518259, Sangon Biotech, Shanghai, China).

The PCR product was ligated with the pMD ^®^ 18-T vector (1000328, TAKARA, Dalian, China). An aliquot of 5 μL of ligation high Solution I, 0.2 μL of pMD ^®^ 18-T vector, 4.8 μL of PCR product, and total volume were combined. After one hour of ligation at 16°C, the resulting product was transformed using the One-step Rapid Competent Cell Preparation Kit (B529307, Sangon Biotech, Shanghai, China). Subsequently, the plasmid was extracted using a SanPrep column plasmid DNA small extraction kit (B518191, Sangon). The constructed plasmid was confirmed by DNA sequencing. The value of plasmid OD_260_ was measured using a microspectrophotometer and converted into copy number (copies/μL).

The standard curve of plasmids were constructed by 10-fold gradient dilution, including 90 μL diluent and 10 μL plasmid. The product was added into X SG Fast qPCR Master Mix (B639271, BBI, Roche, Rotkreuz, Switzerland), and analyzed using quantitative PCR on a LightCycler480 II fluorescence quantitative PCR instrument (Roche, Rotkreuz, Switzerland). The 10 μL PCR reaction mixture included 5 μL of 2X SybrGreen qPCR Master Mix, 0.2 μL of 10 μM primer F, 0.2 μL 10 μM R, 3.6 μL of ddH_2_O, 1.0 μL of DNA. The thermal cycle was 95°C 3min, 95°C 15 s, 60°C 30 s, 45 cycles. The products were placed in a 96-well plate and incubated in LightCycler480 II. After the reaction, the exact copies of *pipme1* gene in *P. infestans* was calculated according to the regression equation established by the standard curve.

### RNA extraction from potato leaves infected by *P. infestans* and reverse transcription

Sporangial suspension of *P. infestans* was inoculated on both sides of potato leaves, and the diseased parts of potato leaves were collected at 0, 12, 24, 36, 48, 60, and 72 h after inoculation, respectively, and frozen in liquid nitrogen and stored at -80°C. RNA was extracted using the UNIQ-10 column Trizol total RNA extraction kit (B511321, Sangon Biotech, Shanghai, China). The integrity and purity of RNA were determined using 1% agarose gel electrophoresis and the ultraviolet spectrophotometer (SMA4000, Merinton Instrument, Inc. Beijing, China). A total of 1200 ng of total RNA was added to the nuclease-free PCR tube on ice, with 1 µL Random Primer p (dN)_6_ (100 pmol), 1 µL dNTP Mix (0.5 mM final concentration), and RNase-free ddH_2_O to bring the volume up to 14.5 µL. The mixture was gently mixed and briefly centrifuged for 3 to 5 s. The reaction mixture was incubated at 65°C for 5 min, ice bathed for 2 min, and centrifuged for 3 to 5 s. A test tube was placed in an ice bath, then 4 µL 5X RT Buffer, 0.5 µL Thermo Scientific RiboLock RNase Inhibitor (20 U), and 1 µL Maxima Reverse Transcriptase (200 U) were added. The tube was gently shaken and centrifuged for 3 to 5 s. A reverse transcription reaction was carried out on a PCR instrument. The obtained cDNA template was used for subsequent expression analysis.

### Real-time relative quantification of *pipme1*

Fluorescence quantitative PCR was performed using cDNA as the template, with the actin housekeeping gene as the target for amplification. PCR primers were actin-F2 (5’ TGCCTGATGGACAAGTTATTACC 3’) and actin-R2 (5’ CCACTGAGCACAATGTTACCG 3’). Fluorescence quantitative real-time PCR (RT-qPCR) was performed using 2X SG Fast qPCR Master Mix (B639271, BBI, Roche) on a LightCycler 480 II fluorescence quantitative PCR instrument. The reaction mixture contained 1.0 μL cDNA, 5 μL SYBR Green qPCR Master Mix, and 0.2 μL 10 μmol/L primers, and 3.6 μL RNase-free water. The thermal cycle consistent with the above. The relative quantitation of gene expression between samples was analyzed using the 2^–ΔΔCT^ method (Livak and Schmittgen, 2001), comparing the cycle threshold (CT) values of the housekeeping gene *actin* and the CT value of the *pipme1* gene.

### Statistical analysis

SPSS version 17.0 software (SPSS Inc., Chicago, IL) and Prism 7.0 software (GraphPad Inc., USA) were used for general statistical analyses. The normality of distribution and homogeneity of variance were evaluated before statistical analysis was conducted. Mean separation among treatments was analyzed by oneway analysis of variance and Duncan’s multiple range test (p < 0.05).

## Results

### Effects of PE on *P. infestans in vitro*

**Figure 1:**
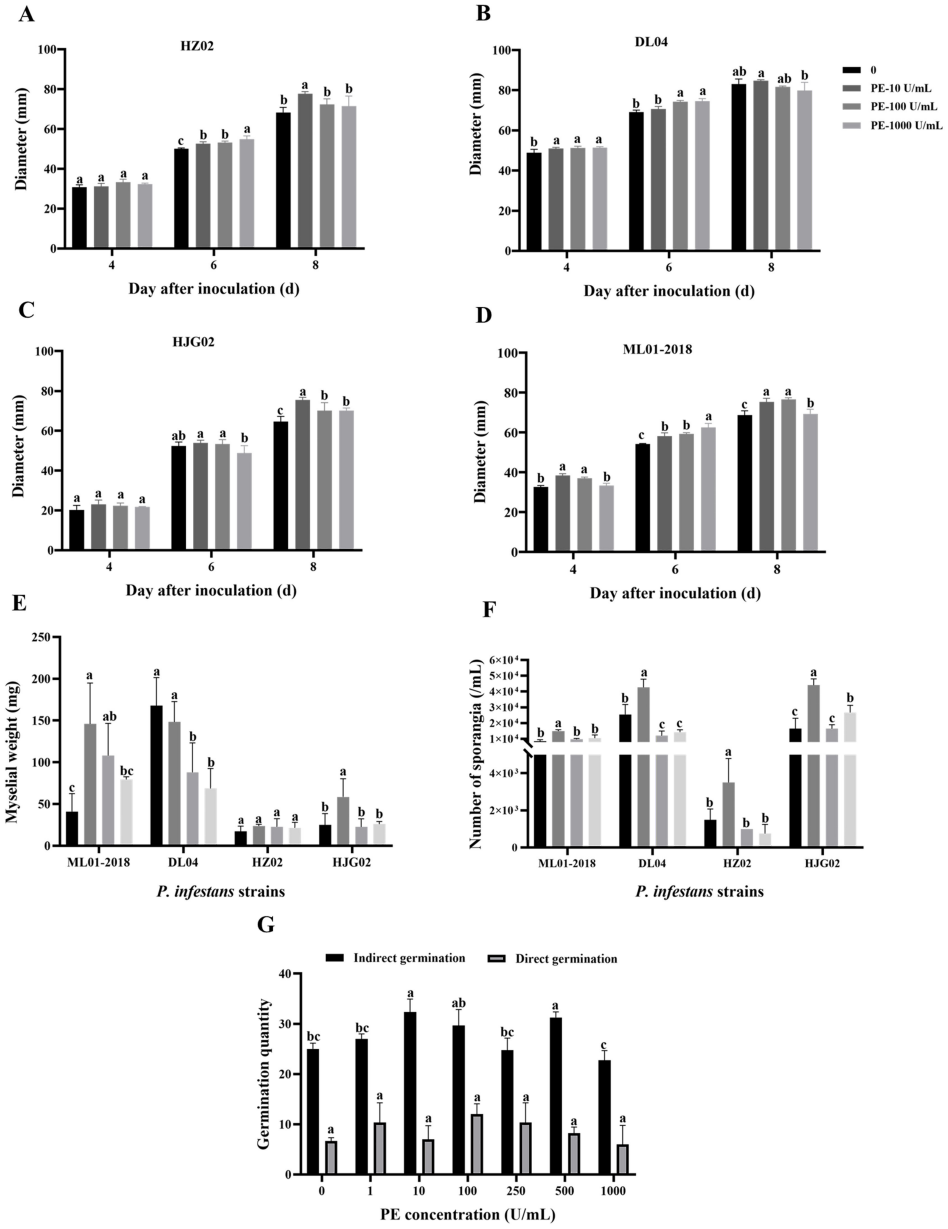
Effects of exogenous addition of pectinesterase (PE) at various concentrations on the colony size of *Phytophthora infestans* strains HZ02 **(A)**, DL04 **(B)**, HJG02 **(C)**, and ML01-2018 **(D)**, mycelial weight of the four strains **(E)**, sporangial production of the four strains **(F)**, and spore germination of DL04 **(G)**. Different letters on the bars indicate significant differences between treatments (*p* < 0.05).

Mycelial weight, sporangia concentration, and spore germination of four strains of *P. infestans* were measured on a PE-amended agar medium. Results showed that PE increased the growth of *P. infestans* to various degrees (**Figure 1**). PE at 10 U/mL significantly increased the weight of mycelia of ML01-2018 and HJG02 (**Figure 1E**). PE at 10 U/mL significantly increased the number of sporangia of all four strains (**Figure 1F**). PE at 10 U/mL and 500 U/mL significantly promoted the sporangial germination but not zoosporic germination of strain DL04 (**Figure 1G**). Therefore, PE promotes the growth of *P. infestans*, increases the weight of mycelium, the number of sporangia, and promotes the sporangial germination of *P. infestans* under certain conditions.

### PE accelerated *P. infestans* infection on potato leaves and tubers

**Figure 2:**
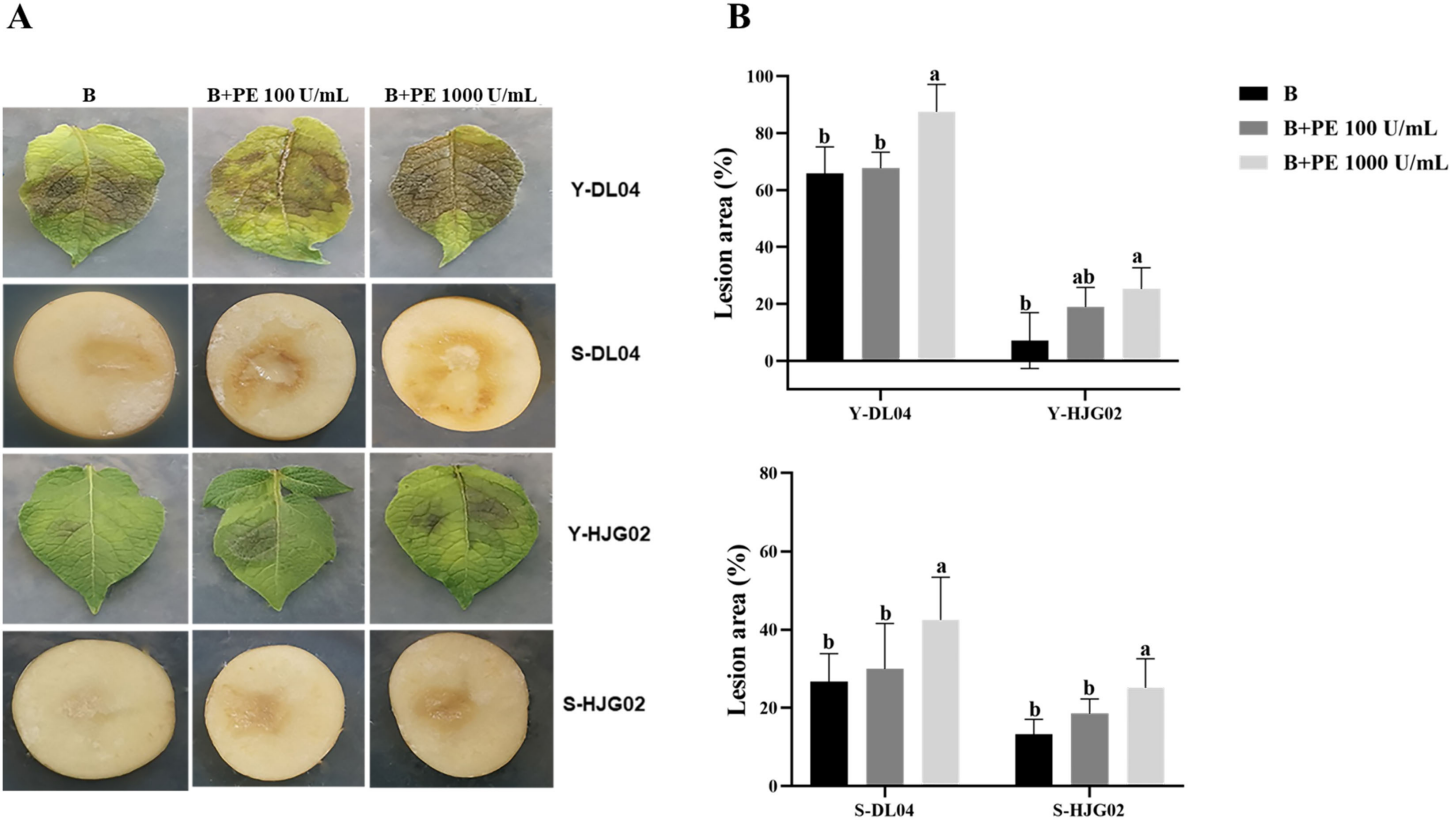
Effects of pectinesterase (PE) treatment and *Phytophthora infestans* DL04 and HJG02 inoculation on potato leaves and tubers. **(A)** Visualization of potato leaf and tuber inoculation; **(B)** Lesion area of infected potato leaves and tubers. B: sporangial suspension of *P. infestans* at 8,000/mL, Y: leaf, S: tuber. Different letters on the bars indicate significant differences between treatments (*p* < 0.05).

Compared with the control, the central lesion area on potato leaves and tubers, inoculated with strains DL04 and HJG02, was largest after adding 1,000 U/mL PE solution, indicating that a high concentration of PE can accelerate the infection of the leaf by *P. infestans* (**Figure 2**). However, this result was not observed at a lower PE concentration (100 U/mL). It is speculated that the pathogenicity of the two strains may be relatively strong, and they can secrete PE themselves. A low concentration of PE is not sufficient to produce a significant infection. It indicated a low concentration of PE had no promoting effect on the infection of potato leaves and tubers by *P. infestans*.

### Measurement of PE activity

**Figure 3:**
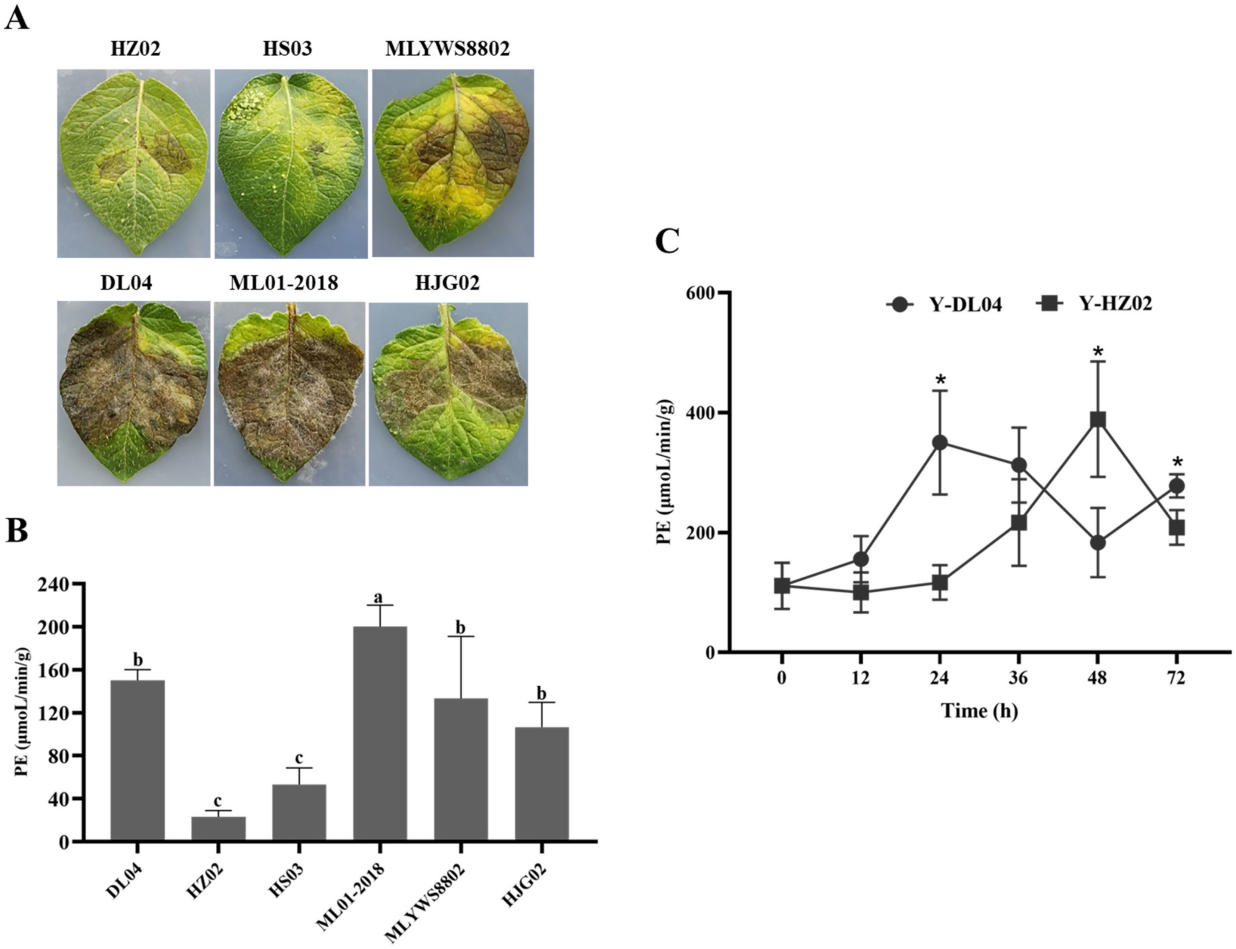
Infection of potato leaves inoculated with *Phytophthora infestans* strains HZ02, DL04, HS03, ML01-2018, MLYWS8802, and HJG02 **(A)**, activities of pectinesterase (PE) in *P. infestans* **(B)**, and enzymatic activities of PE in potato leaves infected by *P. infestans* DL04 and HZ02 **(C)**. Different letters on the bars and the asterisks on the line chart indicate significant differences between treatments (*p* < 0.05).

There were significant differences in PE activity among different *P. infestans* strains after 8 d of culture. Combining with pathogenicity, it was found that ML01-2018 had the strongest pathogenicity, followed by DL04, HJG02 and MLYWS8802; while HZ02 and HS03 had the weakest pathogenicity (**Figure 3A**), the results of PE activity were consistent with the pathogenicity. The correlation between PE activity and pathogenicity of six *P. infestans* was analyzed by Pearson (**Supplementary Figure S1**). There were high positive correlations between the PE activity and pathogenicity of *P. infestans*, the *r* value was 0.94, the linear equation was y = 0.5089x - 8.014 (R^2^ = 0.88, *p* = 0.006), the difference was highly significant (p < 0.01). It indicating that *P. infestans* with high PE activity had strong pathogenicity (**Figure 3B**).

The enzymatic activity in potato leaves inoculated with DL04 and HZ02 was determined at different time points (**Figure 3C**). The enzymatic activity increased with the extension of infection time and decreased after reaching the highest point. The enzymatic activity of DL04 was highest at 24 h after infection, and that of HZ02 was highest at 48 h after infection. The enzymatic activity of DL04 was significantly higher than that of HZ02 at 24 h and 72 h after infection, which was consistent with the pathogenicity results. It indicated that the activity of PE could affect the pathogenicity of *P. infestans* to potato leaves.

### *Pipme1* expression in *P. infestans*

**Figure 4:**
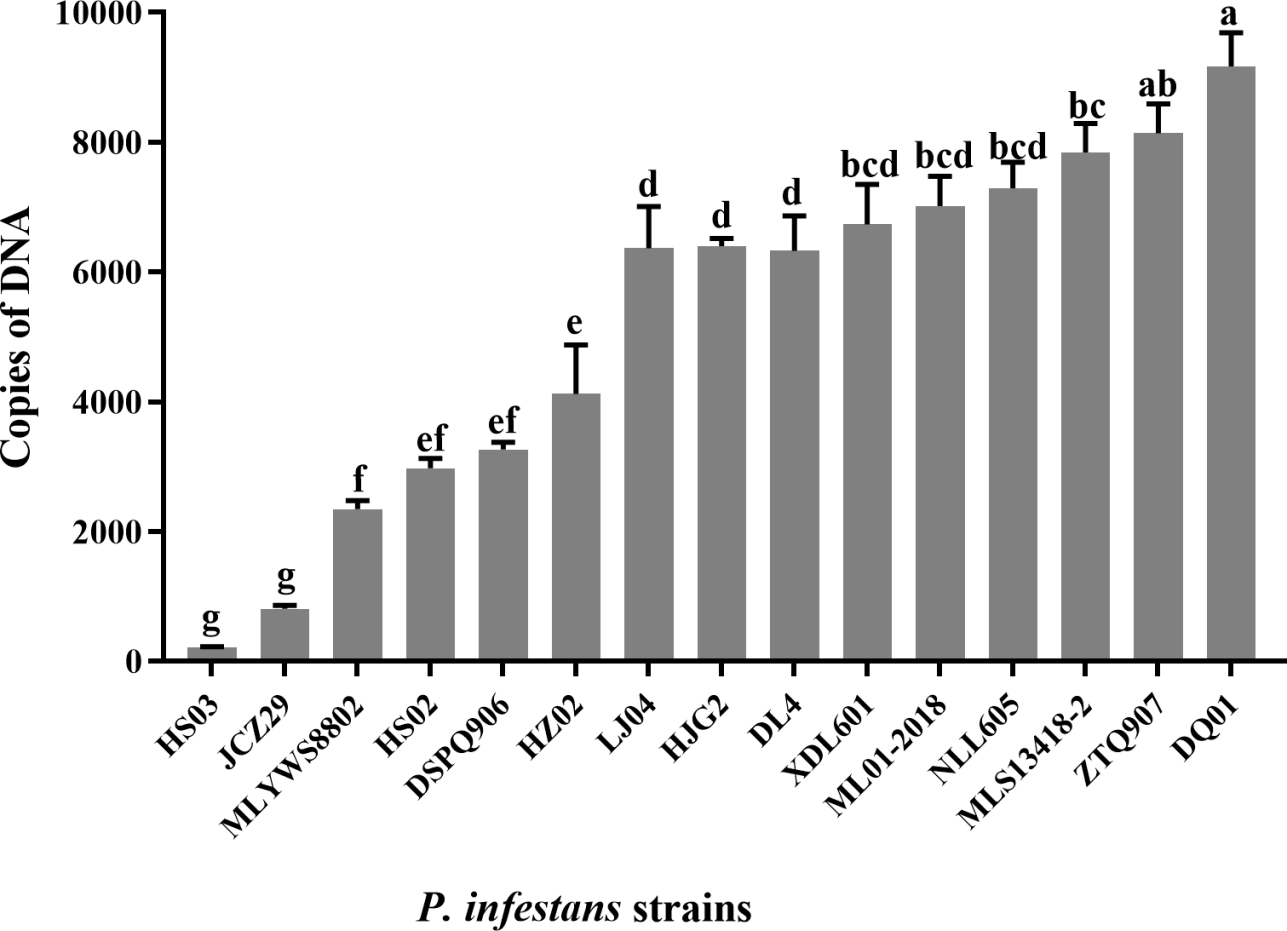
Expression levels (copies of DNA) of the pectinesterase gene in *Phytophthora infestans* strains. Different letters on the bars indicate significant differences between treatments (*p* < 0.05).

PE genes were expressed in different pathogenic strains, and the expression levels of the gene product were significantly different. The gene product level of HS03 and JCZ29 was the lowest (**Figure 4**), while the pathogenicity of four strains of *P. infestans* isolated from Huize, including HS03 and JCZ29, was consistently low. The *pipme1* gene product level of *P. infestans* varied depending on the region of isolation. The level of gene product was in the order from high to low: HS03 (Huize) < MLS13418 (Malong) < LJ04 (Lijiang) < ZTQ907 (Zhaotong) < DQ01 (Diqing). The level of *P. infestans* gene products isolated from the same region was also different. The *pipme1* expression of *P. infestans* strains from Huize varied from high to low: HS03 < JCZ29 < HS02 < HZ02. The level of PE gene product in the strains with strong pathogenicity in the test materials was relatively high. The level of ML01-2018 gene product was higher than that of DL04, HJG02, MLYWS8802, while HZ02 and HS03 had the lowest level, which was consistent with the results of the enzyme activity assay. The correlation between copies of *pipme1* DNA and pathogenicity of 15 strains of *P. infestans* was analyzed (**Supplementary Figure S2**). There were positive correlations between the DNA copies of the pectinesterase gene *pipme1* and pathogenicity, the *r* value was 0.65, the linear equation was y = 0.01x + 10.20 (R^2^ = 0.42; *p* = 0.009), the difference was highly significant (*p* < 0.01). It indicating that the expression of the pectinesterase gene *pipme1* could affect the pathogenicity of *P. infestans* to potato leaves.

### *Pipme1* expression in potato leaves infected by *P. infestans*

**Figure 5:**
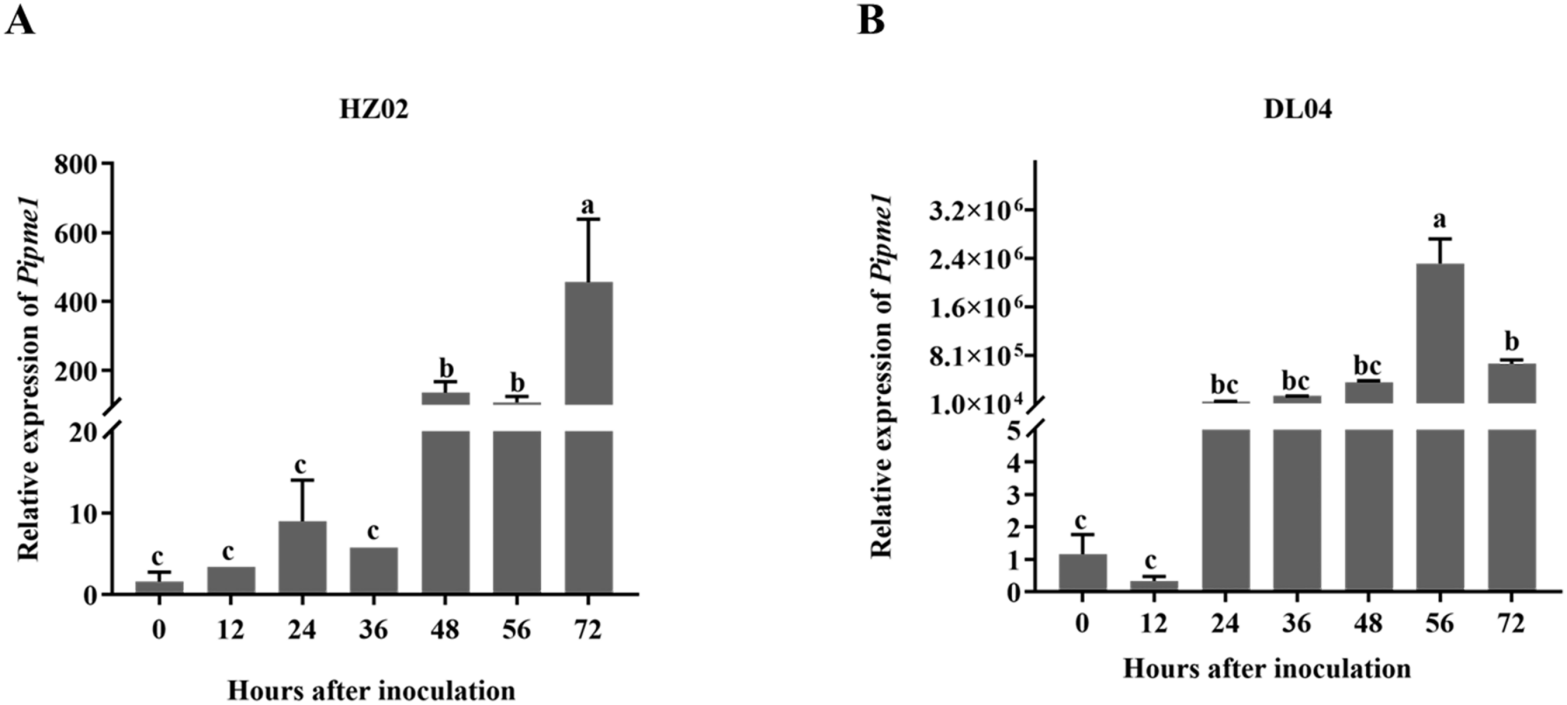
Expression of the pectinesterase gene *pipme1* in potato leaves infected by *Phytophthora infestans* strains HZ02 **(A)** and DL04 **(B)**. Different letters on the bars indicate significant differences between treatments (*p* < 0.05).

The level of PE gene product was determined using fluorescence quantitative PCR in potato leaves infected by *P. infestans* after 0 h, 12 h, 24 h, 36 h, 48 h, 56 h and 72 h. The level of *pipme1* product in HZ02 and DL04 increased with the extension of infection time, which was consistent with the pathogenicity test (**Figure 5**). There was a significant difference in gene product level between HZ02 and DL04 at the same time point after infection. The expression levels of the genes were consistent across different strains. After 48 hours of infection with HZ02, the *pipme1* gene expression was observed, and the expression level increased significantly, reaching the highest at 72 hours post inoculation. The *pipme1* of DL04 began to express at 24 h after infection, and the expression level increased significantly, reaching the highest at 56 h post inoculation. Although the two strains had the same trend of *pipme1* expression after infection, the expression patterns were different. Therefore, the gene expression of DL04 strain with strong pathogenicity was faster than that of HZ02, indicating that the gene was a major factor leading to the pathogenic difference and the speed of leaf disease development.

## Discussion

Plant pathogenic fungi produce multiple cell wall-degrading enzymes, which aid in degrading host cell walls to obtain essential nutrients and facilitate pathogen invasion and colonization, contributing to pathogenicity in plant tissues (Ramezani et al., 2019; Kikot et al., 2009). This activity is a crucial criterion for determining their pathogenicity and defining the infection or pathogenicity of plant pathogens (Esquerre-Tugaye et al., 2000). A higher enzymatic activity correlates with increased pathogenicity in the pathogen (Gawade et al., 2017; Joshi, 2018). Studies have shown that cell wall-degrading enzyme activities vary in *Macrophomina phaseolina* depending on the strains infecting corn, sunflower, and watermelon seeds, leading to different pathogenicity (Ramos et al., 2016). This has also been confirmed in *Colletotrichum gloeosporioides* infecting coffee trees (Armesto et al., 2019) and *Colletotrichum oxysporum* causing rubber tree anthracnose (Fernando et al., 2001). In *Heterobasidion annosum* (Johansson, 1988) and *Pellicularia filamentosa* (Mwenje and Ride, 1997), the pathogenicity is directly related to the activity of their pectinases. By measuring the enzymatic activity in *P. infestans* and *P. infestans*-infected potatoes, we have found that the PE activity is positively correlated with pathogenicity but significantly varied depending on *P. infestans* strains. With the extension of infection time, the PE activity of DL04 was significantly higher than that of HZ02 at 24 h and 72 h. These indicate that pectinesterase activity correlates with pathogenesis of *P. infestans*. Pectinase plays an important role in the early pathogenesis of pathogens (Dallai et al., 2016). For example, the pectin methylesterase *Pcpme6* of *P. capsici* exhibits strong virulence and diversity of transcription when infecting different hosts (Feng et al., 2010). The pectin lyases can induce plant cell death, with *PcPL1*, *PcPL16*, and *PcPL20* being the most aggressive (Fu et al., 2015). Pectin acetylesterase *PlPAE5* of *Peronophythora litchii* plays an significant role in the infection process (Kong et al., 2019). Furthermore, the expression levels of four PG genes vary at different infection stages shown in *C. gloeosporioides* (Jing et al., 2024). In this study, the level of the PE gene *pipme1* expression in *P. infestans* varies significantly with pathogenicity. The expression pattern of *pipme1* after infection is associated with the pathogenicity level, indicating that this gene plays an important role in the progression of late blight. More and more genetic methods are used to study pathogenic factors. The pathogenicity of *Botrytis cinerea* was significantly reduced by knocking out the pectin methylesterase *Bcpme1* gene (Valette-Collet et al., 2003). *Alternaria citri* and *Alternaria alternata* produce polygalacturonases with similar physiological and biochemical characteristics. Knockout of the highly homologous *Acpg1* significantly reduced the pathogenicity of the pathogen, indicating that the virulence of polygalacturonases to the pathogen is related to different pathogenic methods (Isshiki et al., 2001). The site of action will be determined by subcellular localization, and the mechanism of action will be investigated using gene knockout or gene silencing techniques in our future work.

Although PE has been identified as a key factor in the pathogenesis of *P. infestans*, the pathogenesis is complex, because PE is likely one of several cell wall-degrading enzymes contributing to this process (Ramos et al., 2010). Kagda et al. (2020) found that the invertase expression during leaf infection was linked to a decline in apoplastic sucrose, consistent with a role of the enzymes in plant pathogenesis. Hu et al. (2007) found that the mutation of the cellulase gene *eglXoB* in the *Xanthomonas oryzae* pv. *oryzae* PXO99A strain resulted in a decrease in the virulence of the pathogen by about 87% compared to the wild type. Zou et al. (2012) identified the only extracellular protease EcpA from Xoc RS105 strain. After *ecpA* mutation, the protease activity of the strain was completely lost, and the pathogenicity was also significantly reduced compared with the wild type. Hsiao et al. (2010) found that the mannanase activity of Xcc Xc17 strain was completely lost after the mutation of endo-β-mannanase *manA* gene.In this study, we solely focused on PE, excluding other cell wall-degrading enzymes. Therefore, the roles of these enzymes in the pathogenesis of *P. infestans* and their interaction mechanisms require further investigation (Oeser et al., 2002). Further analysis of the redundancy within the multi-gene family will aid in identifying the specificity of different members and exploring their roles in the pathogen-host interactions.

PE can promote the biological characteristics of *P. infestans* and accelerate the infection of *P. infestans* on potato. The pathogenicity of *P. infestans* is related to the enzymatic activity of PE and the expression level of gene *pipme1.* The higher the enzymatic activity and the higher the expression level, the stronger the pathogenicity. Therefore, the pectinesterase activity and expression correlates with pathogenesis of *P. infestans.*

## Data Availability

The original contributions presented in the study are included in the article/**Supplementary Material**. Further inquiries can be directed to the corresponding authors.
