# Supplementary figures and images for: Pectinesterase activity and gene expression correlate with pathogenesis of Phytophthora infestans
Source: Front Plant Sci. 2024 Nov 12;15:1481165. doi: 10.3389/fpls.2024.1481165 (PMC11588465; doi:10.3389/fpls.2024.1481165)

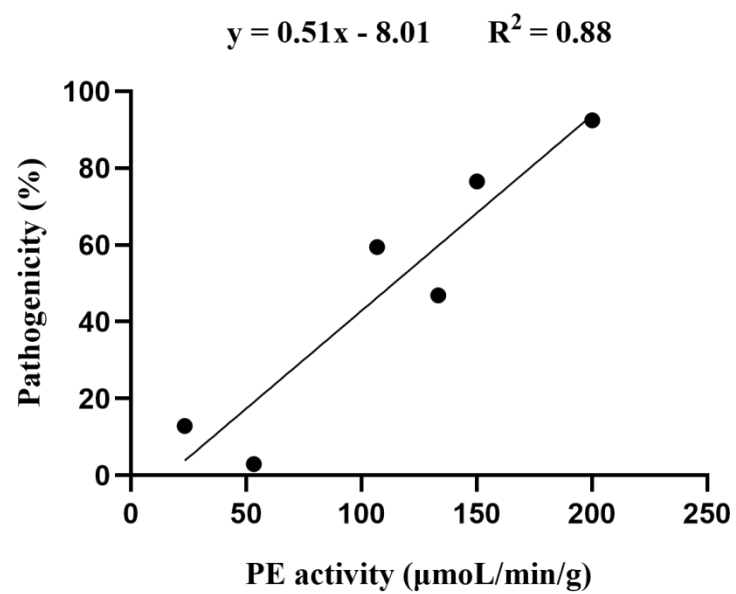

**Figure S1.** The correlation between PE activity and pathogenicity of *Phytophthora infestans*.

Supplement: Supplementary file 1 [file DataSheet1.pdf]
