# Supplementary material for: Pectinesterase activity and gene expression correlate with pathogenesis of Phytophthora infestans
Source: Front Plant Sci. 2024 Nov 12;15:1481165. doi: 10.3389/fpls.2024.1481165 (PMC11588465; doi:10.3389/fpls.2024.1481165)
Supplement: Supplementary file 2 [file DataSheet2.pdf]

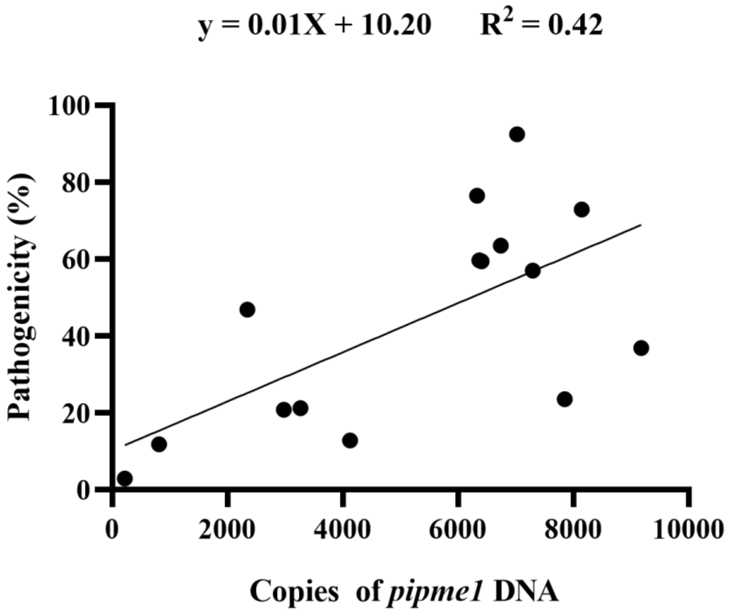

Figure S2. The correlation between Copies of *pipme1* DNA and pathogenicity of *Phytophthora infestans*.
